# Neuronal Control of Adaptive Thermogenesis

**DOI:** 10.3389/fendo.2015.00149

**Published:** 2015-09-25

**Authors:** Xiaoyong Yang, Hai-Bin Ruan

**Affiliations:** ^1^Program in Integrative Cell Signaling and Neurobiology of Metabolism, Yale University School of Medicine, New Haven, CT, USA; ^2^Section of Comparative Medicine, Yale University School of Medicine, New Haven, CT, USA; ^3^Department of Cellular and Molecular Physiology, Yale University School of Medicine, New Haven, CT, USA

**Keywords:** brown adipose tissue, white adipose tissue, beige fat, thermogenesis, hypothalamus, sympathetic nervous system, Ucp1, obesity

## Abstract

The obesity epidemic continues rising as a global health challenge, despite the increasing public awareness and the use of lifestyle and medical interventions. The biomedical community is urged to develop new treatments to obesity. Excess energy is stored as fat in white adipose tissue (WAT), dysfunction of which lies at the core of obesity and associated metabolic disorders. By contrast, brown adipose tissue (BAT) burns fat and dissipates chemical energy as heat. The development and activation of “brown-like” adipocytes, also known as beige cells, result in WAT browning and thermogenesis. The recent discovery of brown and beige adipocytes in adult humans has sparked the exploration of the development, regulation, and function of these thermogenic adipocytes. The central nervous system drives the sympathetic nerve activity in BAT and WAT to control heat production and energy homeostasis. This review provides an overview of the integration of thermal, hormonal, and nutritional information on hypothalamic circuits in thermoregulation.

## A Trio of Fat

Obesity is a rising global epidemic. Excess adiposity is a major risk factor for type 2 diabetes, cardiovascular disease, and hypertension. Overweight and obesity arise when energy intake exceeds energy expenditure and consequently excessive calories are stored in the adipose tissue. The adipose organ comprises white adipose tissue (WAT) and brown adipose tissue (BAT). WAT primarily stores energy as triglycerides, whereas BAT dissipates chemical energy as heat, a process mediated by uncoupling protein 1 (UCP1) (1–3). WAT excess and dysfunction lie at the core of obesity and associated metabolic disorders. By contrast, BAT-mediated adaptive thermogenesis serves to maintain body temperature during cold, contributes to fever during infection, as well as counteracts obesity and related metabolic dysfunction (4). Here, adaptive thermogenesis is defined as non-shivering heat production in response to changes in environmental and physiological settings, such as cold, diet, fever, and stress. Adaptive thermogenesis in BAT is believed to be solely dependent on UCP1 (1–6). However, UCP1-independent thermogenesis in other tissues such as WAT has also been suggested (7, 8).

In 2009, metabolically active BAT was “re-discovered” in adult humans (9–13). The activity of BAT in humans responds to cold challenge and is inversely associated with body mass index (BMI) and age (14). Yet in its infancy, increasing the mass and activity of BAT is considered as a therapeutic option for human obesity.

A “brown-like” type of adipocytes, called beige cells, has been recently discovered in specific WAT depots. Although different from classic brown adipocytes in their origin and molecular identity, beige adipocytes express *Ucp1*, contribute to thermoregulation, and prevent metabolic dysfunction in mice (15, 16). The process of recruiting and activating beige adipocytes is referred to as “browning.” Although classical brown adipocytes are present in adult humans (9, 17, 18), growing evidence suggests that adult human BAT is mainly composed of beige adipocytes (19–21). Considering the fact that interscapular BAT in human infants consists of *bona fide* brown adipocytes and that BAT depots in newborns and adults are located at different sites (22, 23), we speculate that classic brown adipocytes degenerate whereas beige adipocytes gradually prevail with age in humans. Understanding the molecular mechanisms under this transition is important for future therapeutics designed to boost BAT function. It is also intriguing and important to determine the existence and identity of beige adipocytes in other WAT depots, such as subcutaneous fat in humans.

The potential of brown and beige adipocytes as anti-obesity targets attracts extensive interest. The last decade has seen an explosion in our understanding of the development, regulation, and pathophysiology of these distinct adipocytes. Chronic cold exposure, by stimulating the sympathetic nervous system (SNS), is a major and potent activator of BAT thermogenesis and WAT browning. Agonists to β3-adrenergic receptors (AR) that are selectively expressed in brown and beige adipocytes stimulate thermogenesis in both rodents and humans (24). In addition, numerous intrinsic proteins and secreted factors have been shown to affect the development and function of brown and/or beige adipocytes (15, 16, 25). The role of the central nervous system (CNS) in controlling adipose tissue thermogenesis has been an area of intense investigation (26–31). In this review, we summarize the recent progress in our understanding of the central regulation of thermogenesis in brown and beige cells.

## Hypothalamus Orchestrates Metabolism

The hypothalamus acts as to orchestrate homeostatic functions such as food intake, energy expenditure, glucose metabolism, and circadian rhythm. Homeostasis is achieved through the complex crosstalk between the hypothalamus and peripheral tissues in response to environmental cues. The arcuate nucleus (ARC) of the hypothalamus is considered to be a primary integrator of peripheral signals, including hormones and nutrients. Two extensively studied populations of neurons in the ARC are orexigenic neurons expressing agouti-related protein (AgRP)/neuropeptide Y (NPY) and anorexigenic neurons expressing proopiomelanocortin (POMC). These neurons are sensitive, mostly in opposite ways, to hormones such as leptin, insulin, and ghrelin, as well as nutrients such as glucose, amino acids, and fatty acids (32–34). Melanocortin peptides produced by POMC neurons are agonists whereas AgRP is an antagonist of melanocortin-3 and -4 receptors (MC3R and MC4R) that are expressed on the second-order neurons. Located largely in the lateral hypothalamus (LH) and the paraventricular nucleus (PVN) of the hypothalamus, these downstream neurons receive projections from the ARC as well as direct inputs from peripheral signals. Together with the ARC, the LH and PVN function as a metabolic integrator and regulator by projecting to high-order neurons in the CNS and secreting various neuropeptides, for example orexin, melanin concentrating hormone (MCH), cocaine- and amphetamine-regulated transcript (CART), and corticotropin-releasing hormone (CRH).

**Figure 1:**
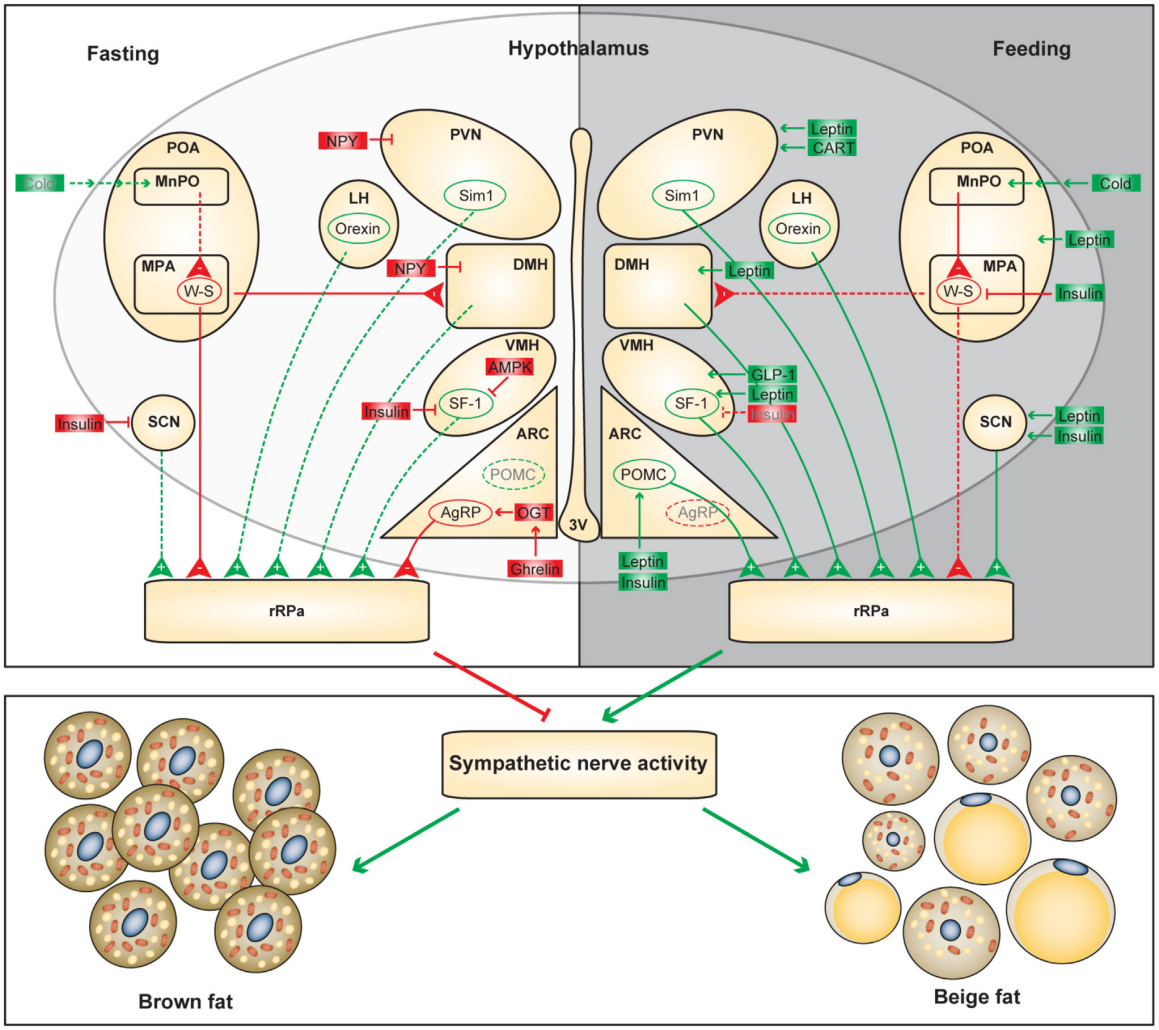
**Functional neuroanatomical model of the hypothalamic control of adaptive thermogenesis**. Differential regulation of the hypothalamic circuits during fasting and feeding is shown. Positive and negative regulators of the adaptive thermogenesis are shown in green and red, respectively. Dashed lines and gray letters indicate inactive or diminished signals.

In addition to their neuroendocrine role, hypothalamic neurons also project to the SNS to control peripheral metabolism (Figure 1). Both BAT and WAT are extensively innervated by the sympathetic fibers that can be tracked back to the hypothalamus (35). By using the neurotropic pseudorabies virus (PRV), a number of studies have described the neuroanatomy of the sympathetic control of adipose tissues in rodents. Although BAT and WAT are anatomically and functionally distinct, common brain areas with efferent projections to both adipose tissues have been identified, including the ARC, LH, and PVN of the hypothalamus and other neuronal sites discussed below (35). Release of catecholamine, particularly norepinephrine (NE), from the sympathetic fibers and subsequent activation of β-AR signaling in adipocytes are necessary for the initiation of lipolysis and the activation of thermogenesis (36). It is thus reasonable to speculate that a “command” neural network dictates these two processes in BAT and WAT (35). It should be noted that only a small portion (about 5–15%) of individual neurons in these common brain regions projects both to BAT and WAT and distinct sympathetic circuits project to different WAT depots (35, 36). It is conceivable that the anatomic architecture of neuronal projections to adipose tissues is evolved to allow differential sympathetic drive across fat depots in response to different lipolytic/thermogenic stimuli (37). Generally speaking, sympathetic drive to BAT is more intense than that to WAT depots, demonstrated by more sympathetic nerve endings on adipocytes, higher NE levels and NE turnover rates, and increased expression levels of tyrosine hydroxylase (37–39). Moreover, there are differential sympathetic activities between various WAT depots at the basal, cold-induced, and fasting-induced conditions (37, 39). It will be interesting to determine what factors control such differential effects of the SNS on adipose tissues.

Along with well-characterized leptin resistance and insulin resistance, catecholamine resistance develops in obesity, which is characterized by impaired catecholamine synthesis and/or sensitivity (40–42). Catecholamine-induced lipolysis and thermogenesis are compromised in WAT of obese animals and patients through several proposed mechanisms, including leptin resistance, α2-AR expression, and inflammation (43, 44). Yet the contribution of catecholamine resistance to the defects in thermoregulation awaits future investigation.

## The Preoptic Area Integrates Thermal Information

Cold exposure and subsequent SNS activation have a profound effect on brown and beige adipocytes. Upon cold exposure, cutaneous transient receptor potential (TRP) cation channels, e.g. TRPM8, sense skin temperature and transmit signals to primary sensory neurons in the dorsal root ganglia (28). This thermal information is delivered to third-order sensory neurons in the lateral parabrachial nucleus (LPB) and then finally to the median preoptic (MnPO) subnucleus of the hypothalamic preoptic area (POA). The medial preoptic area (MPA) of the POA contains warm-sensitive neurons, which receive inhibitory projections from the MnPO (26, 30). Cooling of both distal skin and local hypothalamus reduces the discharge of these warm-sensitive neurons, suggesting that the POA is a central temperature sensor.

Neurons in the POA control SNS activity and thermogenesis by projecting to the dorsomedial hypothalamic area (DMH) and rostral raphe pallidus (rRPa) in the rostral ventromedial medulla, and finally to the sympathetic preganglionic neurons in the intermediolateral nucleus (IML). The neuroanatomical blueprint and neurotransmitters involved in POA regulation of thermogenesis have been reviewed in detail elsewhere (26, 28, 30). Direct cooling of the POA elicits BAT activation (45). Glutamatergic activation of the MnPO and lesions in the inhibitory MPA region both evoke thermogenesis in BAT (46, 47). Conversely, inhibition of MnPO blocks BAT thermogenesis induced by skin cooling (47).

These data demonstrate that the POA integrates thermal information to regulate cold-induced thermogenesis. Febrile responses are also mediated by the POA, which will not be discussed in this review (26, 30). The POA provides efferent signals to WAT depots as well (35); however, roles of the POA in the regulation of WAT browning have not been characterized.

## Hypothalamic Hormone Sensing in Thermoregulation

In the late 1970s, seminal experiments performed by Rothwell and Stock demonstrated that cafeteria diet increased the activity of SNS and BAT (48). Diet-induced thermogenesis has since been considered as an important compensatory mechanism that offsets energy surplus. A series of diets such as cafeteria diet and high-fat diet (HFD) induce metabolic inefficiency and BAT recruitment, thus being called as “recruiting diets” (1). Several factors have been proposed to be important for the thermogenic effect of recruiting diets, including the protein-dilution effect, adipocyte-derived leptin, and hypothalamic neurons (1). Parasagittal hypothalamic knife-cuts and medial hypothalamic lesions in rats impaired cafeteria diet-induced thermogenesis in BAT (49, 50). The PVN was later demonstrated as one of the important hypothalamic nuclei mediating diet-induced thermogenesis (51).

In addition, caloric restriction and daily feeding–fasting cycles also control body temperature and thermogenesis in BAT and beige fat (52, 53, 39). Importantly, cold-induced thermogenesis induces and requires food consumption (39, 54). These data suggest that feeding or sufficient nutrient supply provides permissive signals to adaptive thermogenesis. Leptin secreted by adipocytes in proportion to the fat mass and insulin secreted by pancreatic β cells in response to blood glucose have been proposed as these permissive signals.

### Preoptic area

The POA, as a temperature central sensor, is also under the hormonal regulation. Leptin-responsive neurons are abundant in the POA (55). Cold induces the activity of the leptin receptor (LepR)-expressing neurons in the POA, which project to rRPa to regulate sympathetic BAT inputs (56). The insulin receptor is also detectable in the POA. Insulin injection into the POA decreases the firing rate of warm-sensitive neurons, which results in BAT thermogenesis and elevated core body temperature (57). To understand the molecular mechanisms underlying the integration of various thermogenic cues in the POA will be a promising avenue for future research.

### Arcuate nucleus

The ARC is considered as the primary mediator of leptin signaling in the regulation of energy balance and glucose metabolism (33). Injection of leptin into the ARC increases sympathetic drive to BAT (58), and GABAergic RIP-Cre neurons in the ARC may mediate the ability of leptin to stimulate thermogenesis (59). Orexigenic neuropeptides AgRP and NPY inhibit BAT function, while anorexigenic α-MSH increases SNS activity and BAT function (60, 61).

However, it was not known whether neuronal circuits in the ARC also control WAT browning until recently. Acute activation of hunger-promoting AgRP neurons in the hypothalamus suppresses the browning process. O-linked β-*N*-acetylglucosamine (O-GlcNAc) modification of cytoplasmic and nuclear proteins is a nutrient-sensitive pathway (62, 63). The levels of O-GlcNAc modification and O-GlcNAc transferase (OGT) are enriched in AgRP neurons and are elevated by fasting and the hunger hormone Ghrelin (39). Genetic ablation of OGT in AgRP neurons inhibits neuronal excitability, promotes WAT browning, and protects mice against diet-induced obesity and insulin resistance (39). On the other hand, insulin and leptin act synergistically on POMC neurons to promote WAT browning and prevent diet-induced obesity (64). Phosphatases PTP1B and TCPTP attenuate leptin and insulin signaling in POMC neurons, and double knockout of PTP1B and TCPTP in POMC neurons promotes WAT browning (64). These two complimentary stories demonstrate that the hunger and satiety neurons in the ARC control browning of fat depending on the body’s energy state. Activation of POMC neurons during caloric intake protects against diet-induced obesity whereas activation of AgRP neurons informs the body to store energy during fasting.

### Dorsomedial hypothalamic area

Neurons in the DMH receive GABAergic inputs from warm-sensitive neurons in the MPA (28). Derepression of DMH neurons by infusing an antagonist to the GABA_A_ receptor rapidly and profoundly increases BAT and core body temperature (65, 66), which is dependent on the activation of downstream rRPa neurons (67, 68).

A population of neurons in the DMH expresses the LepR. The intra-DMH injection of leptin increases BAT temperature, in a β3-AR-dependent manner (56, 69). Moreover, blockage of leptin signaling in the DMH blunts the increase in BAT temperature elicited by intraperitoneal injection of leptin (69). Recently, Rezai-Zadeh and colleagues demonstrated that selective activation of DMH neurons by DREADD technique promotes BAT thermogenesis and decreases body weight (70). Conversely, the deficiency of the LepR in the DMH reduces thermogenesis and promotes weight gain (70).

Neuropeptide Y is also expressed by neurons within the DMH, besides the ARC of the hypothalamus. *Npy* expression in the DMH is induced in the conditions where animals demand more energy, for example, chronic food restriction and exercise (71, 72). Similar to that in the ARC, NPY in the DMH promotes food intake and body weight (73). Using viral-mediated knockdown approach in rat, the same group recently showed that DMH knockdown of *Npy* increases BAT activation and the browning of WAT through the SNS (74). Collectively, these data suggest that the DMH is another hypothalamic locus where orexigenic and anorexigenic signals converge to regulate thermogenesis in fat tissue.

### Ventromedial hypothalamic area

The ventromedial hypothalamic area (VMH) is one of the first hypothalamic sites that have been identified to regulate thermogenesis. Although the anatomical linkage between the VMH and adipose tissue is controversial (35), electrical and glutamate stimulation of the VMH has been extensively shown to activate BAT thermogenesis via the SNS (30, 75, 76). Moreover, genetic manipulation of steroidogenic factor-1 (SF-1) neurons, the major population in the VMH, has also demonstrated the importance of the VMH in thermoregulation (77, 78).

Leptin microinjection into the VMH increases glucose uptake in BAT, although thermogenic effect was not tested in the study (79). Selective deletion of the LepR in SF-1 neurons reduces *Ucp1* expression in BAT, suppresses thermogenesis, and produces obesity (80, 81). It has been suggested that the action of leptin on BAT thermogenesis is preferentially mediated by the PI3K/AKT/FOXOI pathway (82, 83). By contrast, insulin suppresses the firing frequency of SF-1 neurons in the VMH (84). Microinjection of insulin to the VMH suppresses BAT thermogenesis in response to cold and glutamate stimulation (85, 86). More interestingly, the magnitude of the suppression shows diurnal rhythm, which is greater at noon than at night in rats (87).

Fatty acid metabolism is essential for neuronal function (88). AMPK-regulated lipogenesis in the VMH is an important regulator of BAT thermogenesis and controller of body weight (89). Recently, Lopez and colleagues further showed that various hormones, including thyroid hormones, estradiol, and glucagon-like peptide-1 (GLP-1), activate BAT thermogenesis through relieving the suppression on lipogenic genes by AMPK in the VMH (89–91). These studies strongly argue that the VMH senses hormonal cues to regulate BAT function; however, the involvement of the VMH in the regulation of WAT browning has not been explored.

### Paraventricular nucleus

The PVN is strongly susceptible to trans-synaptic infection by RPV from BAT (35); however, its effect on thermogenesis has been controversial. Electrical stimulation of the PVN does not affect BAT function (92). Microinjection of *N*-methyl-d-aspartate (NMDA) or bicuculline to activate neurons in the PVN blocks sympathetic drive to BAT induced by cold, indicating that the PVN negatively regulates thermogenesis (93). Paradoxically, stimulation of the PVN with glutamate is shown to activate BAT thermogenesis (94).

There is a large population of MC4R-expressing neurons in the PVN, and the MC4R agonist melanotan II (MTII) induces BAT thermogenesis (95). Single-minded 1 (Sim1) is necessary for development of the PVN, and ablation of Sim1 neurons reduces *Ucp1* expression, BAT temperature, and energy expenditure (96). Restoration of MC4R in the Sim1 neurons fails to rescue reduced energy expenditure in *Mc4r* knockout mice, suggesting that MC4R elsewhere controls energy expenditure (97). However, a recent study of *Mc4r* deletion in Sim1 neurons showed that Sim1 neurons are important in the regulation of energy expenditure (98).

Leptin regulates the synaptic activity of neurons in the PVN that project to BAT (99). Viral administration of leptin in the PVN stimulates the expression of *Ucp1* in BAT (100). The orexigenic peptide NPY reduces energy expenditure; however, its site of action was not identified until recently (101). Shi et al. demonstrated that ARC-derived NPY decreases tyrosine hydroxinase (TH) expression in the PVN to down-regulate BAT *Ucp1* expression and energy expenditure (61). On the other hand, the anorexigenic peptide CART, when injected in the PVN, induces the *Ucp1* expression in both BAT and WAT (102).

### Suprachiasmatic nucleus

Core body temperature, like all vital aspects of physiology and metabolism, shows circadian rhythm (103). Thermogenic plasticity in BAT is also rhythmic and under the control of cell-autonomous clock machinery (104–107). Directly entrained by light, the suprachiasmatic nucleus (SCN) in the hypothalamus has been established as a circadian pacemaker that synchronizes peripheral clocks across the body to adjust behavior and physiology in accordance with the day/night cycle (108, 109). Both BAT and WAT receive sympathetic flow that can be tracked back to the SCN (35, 110). Glutamate injection to activate the neurons in the SCN stimulates BAT thermogenesis, and this effect is greater during the dark phase (111). Nevertheless, it is still unknown whether the SCN controls the circadian rhythm of thermogenesis and if WAT browning is also under circadian regulation.

LepR-expressing neurons exist in the SCN, and leptin phase-advances the circadian rhythm of SCN on brain slices (112). *In vivo* analysis revealed that leptin modulates clock gene expression in the SCN and the sleep/wake cycle (113). It will be of interest to determine whether leptin in the SCN regulates the circadian rhythm of thermogenesis. In addition, intra-SCN injection of insulin to rats decreases the sympathetic activity on BAT at noon (fasted state) and increases the activity at night (fed state), indicating a circadian control of BAT function by insulin action on the SCN (87). These data suggest that the SCN may receive peripheral satiety cues to modulate the circadian rhythm of energy expenditure to maintain homeostasis.

## Psychological Regulation of Thermogenesis

Psychological fever, one of the most common psychological diseases, is characterized by acute or persistent increase in body temperature when patients are psychologically stressed (101). It was suggested that SNS-activated BAT thermogenesis is responsible for stress-induced hyperthermia (114, 115). Orexin neurons in the hypothalamus control multiple physiological processes, including arousal, wakefulness, and appetite. The orexin neurons are considered important for inflammatory fever and the defense against cold (116). It has also been shown that the orexin neurons are indispensable for the stress-induced *Ucp1* expression and thermogenesis in BAT and resultant hyperthermia (117). Recently, a functional neuroanatomical study using an optogenetic approach has identified a DMH-medullary raphe circuit that drives psychological stress-activated BAT thermogenesis and hyperthermia (118).

Environmental enrichment is the stimulation of the brain by physical and social surroundings. This regimen supports neurogenesis and could aid the treatment of neurodegenerative diseases (119). Intriguingly, environmental enrichment in rodents induces WAT browning and decreases adiposity (120). Hypothalamic brain-derived neurotrophic factor (BDNF) has been shown to increase thermogenesis and energy expenditure by acting on neurons in the PVN and VMH (121, 122). Environmental enrichment up-regulates hypothalamic BDNF, and the inhibition of BDNF blocks the environmental enrichment-induced WAT browning (120).

## Summary and Perspective

Yet to be fully defined, the developmental and functional identities of brown and beige adipocytes are distinct (16). Separate populations of neurons in common areas of the hypothalamus projecting to BAT and different WAT depots may contribute to the functional and regulatory differences between fat depots (35). The blueprint of the neuroanatomical regulation in BAT thermogenesis is becoming apparent; however, we just begin to explore the central regulation of the browning process in WAT (39, 64, 123).

It is not surprising that the hypothalamic areas that control food intake also modulate adaptive thermogenesis. In the hunger state, orexigenic neurons attenuate SNS-mediated heat production in brown and beige fat to preserve energy; conversely, in the satiety state, anorexigenic neurons promote energy expenditure to maintain homeostasis (Figure 1). However, adaptive thermogenesis is not efficacious to offset long-term energy imbalance, which partially explains the susceptibility to diet-induced obesity and the difficulty of weight loss by dietary interventions in humans. It is important to unravel the biological logic of distinct hypothalamic efferent outputs. In addition to mapping the neuronal circuits controlling thermogenesis in BAT and WAT, there is an urgent need to identify intracellular mechanisms by which neuronal activities are dictated by thermogenic cues. Understanding the molecular and cellular basis of neuronal regulation of adaptive thermogenesis will lay a foundation for future therapeutics against obesity. For example, GLP-1 receptor agonists, which have been widely used to treat diabetes, can be administrated centrally to stimulate brown adipose thermogenesis (90). ARC-specific administration of OGT inhibitors and PTP1B inhibitors suppress AgRP neurons and stimulate POMC neurons, respectively, to promote energy expenditure in brown and beige adipocytes (39, 64).

Hypothalamic circuits respond to both thermal information and nutritional cues to modulate cold- and diet-induced thermogenesis. It has been demonstrated that feeding provides permissive signals for the activation of brown and beige adipocytes upon cold challenge (39, 54). It is conceivable that yet-to-be-found neuronal circuits exist in the hypothalamus serve as an integrator of various thermogenic stimuli. Characterization of such integration sites will help us understand how mammals, including human beings, tradeoff between life-history variables such as hunger and cold during the evolution.

Adaptive thermogenesis is thought to evolve slowly (in days) to adjust to changes in temperature and food availability/composition; however, the activation or silencing of CNS and SNS respond immediately (in minutes) to stimuli, followed by delayed (in hours) changes in *Ucp1* expression. Appropriate tools to manipulate neurons should be utilized to better characterize neuronal networks in thermoregulation. Studies using the chemical/electrical stimulation and anatomical lesions are difficult to control efficacy and often lead to off-target effects. Genetically modified animal models can continue to be used to reveal neuron population-specific function. However, the caution should be taken because genetically modified animals may exhibit developmental defects due to non-specific Cre expression and observed phenotypes are often the results of the long-term and/or secondary effects. Recent advances in optogenetics and chemogenetics produce powerful tools to acutely and precisely control neuronal activity (124). These tools in combination with classical transgenic approaches will accelerate the progress in defining the functional neuronal architecture in thermoregulation.

## Conflict of Interest Statement

The authors declare that the research was conducted in the absence of any commercial or financial relationships that could be construed as a potential conflict of interest.
